# Mast cell MrgprB2 in neuroimmune interaction in IgE-mediated airway inflammation and its modulation by β-arrestin2

**DOI:** 10.3389/fimmu.2024.1470016

**Published:** 2024-10-17

**Authors:** Sangita Sutradhar, Hydar Ali

**Affiliations:** Department of Basic and Translational Sciences, School of Dental Medicine, University of Pennsylvania, Philadelphia, PA, United States

**Keywords:** allergic airway inflammation, β-arrestin2 (β-arr2), mast cells, Mas-related G protein coupled receptor B2 (MrgprB2), substance P

## Abstract

**Introduction:**

Allergic asthma has been linked to the activation of mast cells (MCs) by the neuropeptide substance P (SP), but the mechanism underlying this neuroimmune interaction is unknown. Substance P produced from cutaneous nociceptors activates MCs via Mas-related G-protein-coupled receptor B2 (MrgprB2) to enhance type 2 immune response in experimental atopic dermatitis in mice. We recently showed that the adapter protein β-arrestin2 (β-arr2) contributes to MrgprB2-mediated MC chemotaxis. The goals of this study were to determine if MrgprB2 facilitates neuroimmune interaction in IgE (FcεRI)-mediated allergic airway inflammation (AAI) and to assess if this response is modulated by β-arr2.

**Methods:**

Wild-type (WT), *MrgprB2^−/−^* mice and mice with MC-specific deletion of β-arr2 (*Cpa3^Cre+^*/*β-arr2^fl/fl^*) were passively sensitized with anti-TNP-IgE and challenged with antigen. The generation of SP and MC recruitment in the lung were determined by immunofluorescence and toluidine blue staining, respectively. The transcripts for Tac1, MrgprB2, TNF-α, and Th2 cytokines in lung tissue were assessed by RT-PCR, and the release of selected cytokines in bronchoalveolar lavage (BAL) was determined by ELISA. Eosinophil and neutrophil recruitment in lung tissue and BAL were determined by immunofluorescence staining and flow cytometry, respectively. Goblet cell hyperplasia was determined by periodic acid–Schiff staining.

**Results:**

Following IgE sensitization and antigen challenge in WT mice, SP generation, and MC recruitment, transcripts for Tac1, MrgprB2, TNF-α, and Th2 cytokine were upregulated when compared to the control challenge. TNF-α, Th2 cytokine production, eosinophil/neutrophil recruitment, and goblet cell hyperplasia were also increased. These responses were significantly reduced in *MrgprB2^−/−^* and *Cpa3^Cre+^*/*β-arr2^fl/fl^* mice.

**Discussion:**

The data presented herein suggest that SP-mediated MrgprB2 activation contributes to AAI and goblet cell hyperplasia in mice. Furthermore, these responses are modulated by β-arr2, which promotes MC recruitment to facilitate their activation through FcεRI.

## Introduction

Mast cells (MCs) express high-affinity IgE receptor (FcεRI) and are widely recognized as a key player in atopic dermatitis (AD) and allergic asthma by mediating effector function in type 2 immune response-driven inflammation (1–6). In addition to FcεRI, a subtype of human MCs expresses a G-protein-coupled receptor (GPCR) known as MRGPRX2 (mouse ortholog MrgprB2) (7, 8). MCs are found near peripheral nerve endings in most tissues and act as the first responder to sensory nerve activation (9, 10). There is evidence to suggest that neuroimmune interaction is a key modulator in the manifestation of pathological outcomes in AD, ulcerative colitis, and pain through the activation of MCs by substance P (SP) via MrgprB2 (5, 10, 11).

In addition to MCs, airway sensory neurons express FcεRI, and its activation results in increased expression of the *Tac1* and the release of its gene product SP, which serves to amplify type 2 allergic airway inflammation (AAI) in mice (12). Human MCs are traditionally subdivided into two categories based on the composition of their secretory granules. MCs that contain tryptase, chymase, cathepsin G, and carboxypeptidase A3 (CPA3) are known as MC_TC_ (13–17). By contrast, MCs containing only tryptase are known as MC_T_ (16). The original demonstration that the MC_T_ type of MCs found in the lung do not respond to SP for degranulation called into question their relevance in neuroimmune interaction in AAI (14). However, the level of SP is elevated in lungs, bronchoalveolar lavage (BAL), and sputum obtained from asthmatic patients compared to normal controls, and these MCs undergo degranulation in response to SP (18–20). A more recent study revealed that ~12% of normal human lung MCs express MRGPRX2 (17). Furthermore, in individuals with severe asthma, airway submucosa and lung epithelium are dominated by MC_TC_ rather than MC_T_ (21–23). We recently demonstrated that the number of MRGPRX2-positive MCs is significantly enhanced in the lungs of the patients who died from asthma compared to patients who died from unrelated causes (24). However, the possibility that neuroimmune interaction involving MrgprB2 expressed in MCs contributes to AAI in mice has not been tested.

The recruitment of inflammatory cells in AAI is mediated through the activation of GPCRs for chemokines and proteases (25–27). In addition to G-proteins, most GPCR agonists activate downstream signaling pathways that involve the recruitment of adapter proteins known as β-arrestin (β-arr) (28). GPCR agonists that preferentially activate either G-proteins or β-arrs are known as G-protein biased and β-arrestin biased, respectively. However, agonists that activate both pathways are known as balanced agonists. β-arr-biased signaling was initially characterized for its role in GPCR desensitization, but it also provides downstream signaling for chemokine generation and cell migration (29–33). Thus, chemokine- and protease-induced Th2 cell Chemotaxis require the presence of β-arr2 (34–36). Furthermore, in models of allergic asthma, *β-arr2^−/−^* mice display reduced type 2 inflammation and mucin secretion (36–38). It has been proposed that β-arr2 biased signaling for type 2 inflammation could provide a novel target for modulating AAI (27, 38). We have recently shown that β-arr2 promotes MrgprB2 signaling for MC chemotaxis (39). However, the possibility that β-arr2 modulates AAI has not been determined.

For the present study, we utilized *MrgprB2^−/−^* and mice with MC-specific deletion of β-arr2 (*Cpa3^Cre+^*/*β-arr2^fl/fl^*) to determine their roles in IgE-mediated AAI and mucus secretion. The data presented herein provide novel crosstalk between FcεRI and MrgprB2 and suggest that targeting MrgprB2’s β-arr2-biased signaling could provide a new approach for modulating AAI.

## Materials and methods

### Reagents

Anti-TNP IgE (Cat: 557079, 0.5 mg/mL) and Rat Anti-Substance-P (556312) were purchased from BD Pharmingen (Franklin Lakes, NJ, USA). TNP-Ova (sc-396493) was purchased from ChemCruz (Dallas, TX, USA). Hank’s balanced salt solution (HBSS), 0.5 M ethylenediaminetetraacetic acid (EDTA), and type IV collagenase (17104-019) were purchased from Life Technologies Corporation (Grand Island, NY, USA). DNase I (10104159001) was purchased from Roche Diagnostics (Mannheim, Germany). Dispase II (D4693-1G), periodic acid–Schiff reagent (395B-1KT), and toluidine blue (T3260-5G) were purchased from Sigma Aldrich (St. Louis, MO, USA). ACK lysing buffer (#118-156-721) was purchased from Quality Biological (Gaithersburg, MD, USA). All flow cytometry antibodies including Zombie Yellow (77168), CD16/32 (clone 93, 101320), CD45-pacific blue (clone 30-F11, 103126), CD11b-per CP-cy5.5 (clone M1/70, 101228), Ly6G-FITC (clone 1A8, 127606), CD170-PE (Siglec-F, clone S17007L, 155506), and Fixation Buffer (#420801) were purchased from BioLegend (San Diego, CA, USA). UltraComp eBeads (01-3333-42), Alexa Fluor 647-conjugated donkey anti-mouse IgG (A31571), Alexa Fluor 647-conjugated donkey anti-rat IgG (A48272), and Alexa Fluor 488-conjugated goat anti-rabbit IgG (A11008) secondary antibodies were purchased from Invitrogen (Carlsbad, CA, USA). Protein Gene Product (PGP) 9.5 rabbit polyclonal antibody (GTX109637) was purchased from GeneTex (Irvine, CA, USA). Human/mouse myeloperoxidase (MPO) and polyclonal goat IgG antibody (AF-3667-SP) were purchased from R&D Systems (Minneapolis, MN, USA). Anti-mouse monoclonal eosinophil peroxidase (EPx) antibody (clone MM25-8.2.2) was obtained from Mayo Clinic, Phoenix, AZ, USA. Alexa Fluor 647-conjugated donkey anti-goat IgG (705-605-003) and Alexa Fluor 488-conjugated donkey anti-rat (712-545-150) antibodies were purchased from Jackson ImmunoResearch Laboratories (West Groove, PA, USA). Rodent block M (RBM961g) was purchased from BioCare Medical (Pacheco, CA, USA). DAPI and ProLong Gold antifade reagent were purchased from Molecular Probes (Eugene, OR, USA). RNeasy Fibrous Tissue Kit was purchased from Qiagen (Hilden, Germany). High-Capacity RNA-to-cDNA Kit (#4368814) and Fast SYBR Green Master mix (#4385612) were purchased from Applied Biosystems (Vilnius, Lithuania).

### Mice

Mice were kept in a pathogen-free environment and on hardwood bedding that had been autoclaved. All investigations were carried out on 8–10-week-old mice of both genders. CRISPR/Cas9 technology was used by the CRISPR core at the University of Pennsylvania to develop *MrgprB2^−/−^* mice as described previously (40), and C57BL/6 [wild-type (WT)] mice were obtained from the Jackson Laboratory (Bar Harbor, ME, USA). β-arr2*^flox/flox^* (*β-arr2^fl/fl^*) mice were a kind gift from Robert Lefkowitz and Julia Walker (Duke University Medical Center, Durham, NC, USA); *Cpa3-Cre* (Cre is expressed under the promoter of Cpa3, expressed only in MCs) mice were generously provided by Stephen J. Galli (Stanford University, Stanford, CA, USA). As previously described, animals with an MC-specific deletion of β-arr2 (*Cpa3^Cre+^*/*β-arr2^fl/fl^*) were produced by mating *Cpa3Cre* mice with *β-arr2^fl/fl^* mice (41). The University of Pennsylvania’s Institutional Animal Care and Use Committee granted approval (Protocol Number 803883) for the use of mice.

### Induction of lung inflammation

Lung inflammation was induced in the experimental model by modifying the protocol of Jin et al. (6). Mice were anesthetized using 50 µL of ketamine–xylazine intraperitoneally and were passively sensitized with 10 µg of anti-TNP IgE in 100 µL phosphate-buffered saline (PBS) through retro-orbital injection. After 24 hours, mice were anesthetized, and 200 µg of antigen (TNP-Ova) or PBS was administered intranasally. After 24 hours of intranasal challenge, mice were sacrificed and used for experiments, as described below.

### Collection of bronchoalveolar lavage

The tracheas of individual mice were exposed and cannulated. To obtain BAL, lungs were instilled with 1 mL of 1× HBSS with 100 µM EDTA three times (6). The first flush was kept separately to collect cell-free supernatant for ELISA and stored at − 80°C.

### Histology

Lung samples from experimental mice were fixed in 10% formalin overnight. Samples were dehydrated through a series of increasing ethanol washes and embedded in paraffin. Paraffin-embedded samples were sliced into 5 μm cross sections and stained with toluidine blue as described previously (42). Quantitation of MC was conducted by counting the number in a total of five fields from different lung tissue sections of both control and challenged mice, and the mean was calculated in each mouse. The lung sections were also stained with H&E.

Periodic acid–Schiff (PAS) staining was performed according to the manufacturer’s protocol. Briefly, sections were deparaffinized and hydrated. The slides were immersed in periodic acid solution for 5 min, rinsed and immersed in Schiff’s reagent for 15 min at room temperature (RT), washed and counterstained with hematoxylin, and washed. Finally, the slides were dehydrated and mounted in xylene-based mounting media. Images were captured by Nikon Eclipse Ni microscope (Minato City, Tokyo, Japan) using ×20 magnification.

### Flow cytometry

Single-cell suspension was prepared by digesting lung tissue in RPMI10 media containing type IV collagenase (600 U/mL), dispase II (1 mg/mL), and DNase I (25 μg/mL) for 30 min at 37°C. The digested tissue was filtered through a 70-μm filter, washed, and suspended in FACS buffer [PBS containing 2% fetal bovine serum (FBS)]. Each sample was Fc blocked with anti-CD16/32 antibody (1:100) for 15 min at 4°C. Samples were then stained with respective antibody cocktail (neutrophils —Zombie Yellow, CD45, CD11b, and Ly6G; eosinophils —Zombie Yellow, CD45, CD11b, and CD170) for 30 min at 4°C. Samples were washed and then fixed with Fixation Buffer. Data were acquired using a BD LSR II flow cytometer (San Jose, CA, USA) and analyzed using the FlowJo software version 10.8.1 (Tree Star Inc., Ashland, OR, USA). For single stain compensation, beads (UltraComp eBeads) were used (43).

### Immunofluorescence staining

Immunofluorescence experiments were performed using the same tissue blocks as used in histological studies. EPx staining was conducted following the protocol as described previously (44). Briefly, tissue sections were deparaffinized, hydrated, antigen retrieved, and incubated with pepsin for 30 min at room temperature. The sections were then washed, blocked with rodent block M, and incubated with mouse anti-EPx (1:50) primary antibody overnight at 4°C. Sections were then washed three times with PBS containing 0.1% Tween 20 and incubated with Alexa Fluor 647-conjugated donkey anti-mouse IgG secondary antibodies (1:750). The tissue sections were then washed and mounted with DAPI.

Neutrophil (MPO), substance P, and PGP9.5 staining were performed as described previously, with some modifications (45–47). Briefly, tissue sections were deparaffinized, hydrated, antigen retrieved, and blocked with 1% bovine serum albumin (BSA) in PBS-T (PBS containing 0.2% Triton X-100). Tissue sections were incubated with human/mouse MPO polyclonal goat IgG (1:250), rat anti-SP (1:200), and PGP9.5 rabbit polyclonal (1:250) primary antibodies overnight at 4°C. Sections were then washed three times with PBS and incubated with Alexa Fluor 647-conjugated donkey anti-goat IgG (1:1,500), Alexa Fluor 488-conjugated donkey anti-rat IgG (1:500), Alexa Fluor 647-conjugated donkey anti-rat IgG (1:1,500), and Alexa Fluor 488-conjugated goat anti-rabbit IgG (1:750) for 1 hour at room temperature in the dark. The tissue sections were then washed and mounted with DAPI. Images were acquired using a Nikon Eclipse Ni microscope.

### Quantitative real-time PCR

Lung samples were homogenized, and total RNA was extracted and purified by RNeasy Fibrous Tissue Kit. A total of 1 µg of RNA was used to synthesize cDNA using the High-Capacity RNA-to-cDNA Kit according to the manufacturer’s instructions. All the qRT-PCRs were performed using Fast SYBR Green Master Mix. Using specific primers, the mRNA expressions of mouse *MrgprB2* (forward 5′-TACTTCTGCAGAGAGCCATGC-3′, reverse 5′-GCTGCAGCTCTGAACAGTTTC-3′), *TNF-α* (forward 5′-TCTCATGCACCACCATCAAGGACT-3′, reverse 5′-ACCACTCTCCCTTTGCAGAACTCA-3′), *IL-4* (forward 5′-TCCTCACAGCAACGAAGAACACCA-3′, reverse 5′-GCAGCTTATCGATGAATCCAGGCA-3′), *IL-5* (forward 5′-CTCTGTTGACAAGCAATGAGACG 3′, reverse 5′-TCTTCAGTATGTCTAGCCCCTG-3′), *IL-13* (forward 5′-AGACCAGACTCCCCTGTGCA-3′, reverse 5′-TGGGTCCTGTAGATGGCATTG-3′), *Tac1* (forward 5′-GGCCAAGGAGAGCAAAGA-3′, reverse 5′-CGAGGATTTTCATGTTCGATT-3′), *Tac4* (forward 5’-GTAGCTTCCTCAGCCATGCAG-3, reverse 5’-CCGCCCCCAAATACAATACA-3’), and *Actin* (forward 5′-GATTACTGCTCTGGCTCCTAGC-3′, reverse 5′-GACTCATCGTACTCCTGCTTGC-3′) were analyzed using QuantStudio 3 Real-Time PCR System (Thermo Fisher Scientific, Waltham, MA, USA). The analysis was performed in triplicate, and Actin was used as an internal control. All data presented as fold changes of target genes were determined using the 2^−ΔΔCt^ method with actin as a stable reference gene.

### Cytokine assays

Mouse BAL TNF-α and IL-13 were assessed using immunoassay kits (R&D Systems, Minneapolis, MN, USA) according to the manufacturer’s instructions. The limits of detection for each enzyme-linked immunosorbent assay (ELISA) were 5–10 pg/mL.

### Statistical analysis

Data shown are mean ± SEM values derived from at least three independent experiments. Statistical significance was determined by two-way ANOVA. Error bars represent mean ± SEM from at least three independent experiments. Significant differences were set at *p ≤ 0.05, **p ≤ 0.01, ***p ≤ 0.001, and ****p ≤ 0.0001 and analyzed using GraphPad Prism, version 6.07.

## Results

### MrgprB2 contributes to MC recruitment and SP upregulation in the lung of IgE sensitization and antigen-challenged mice

**Figure 1:**
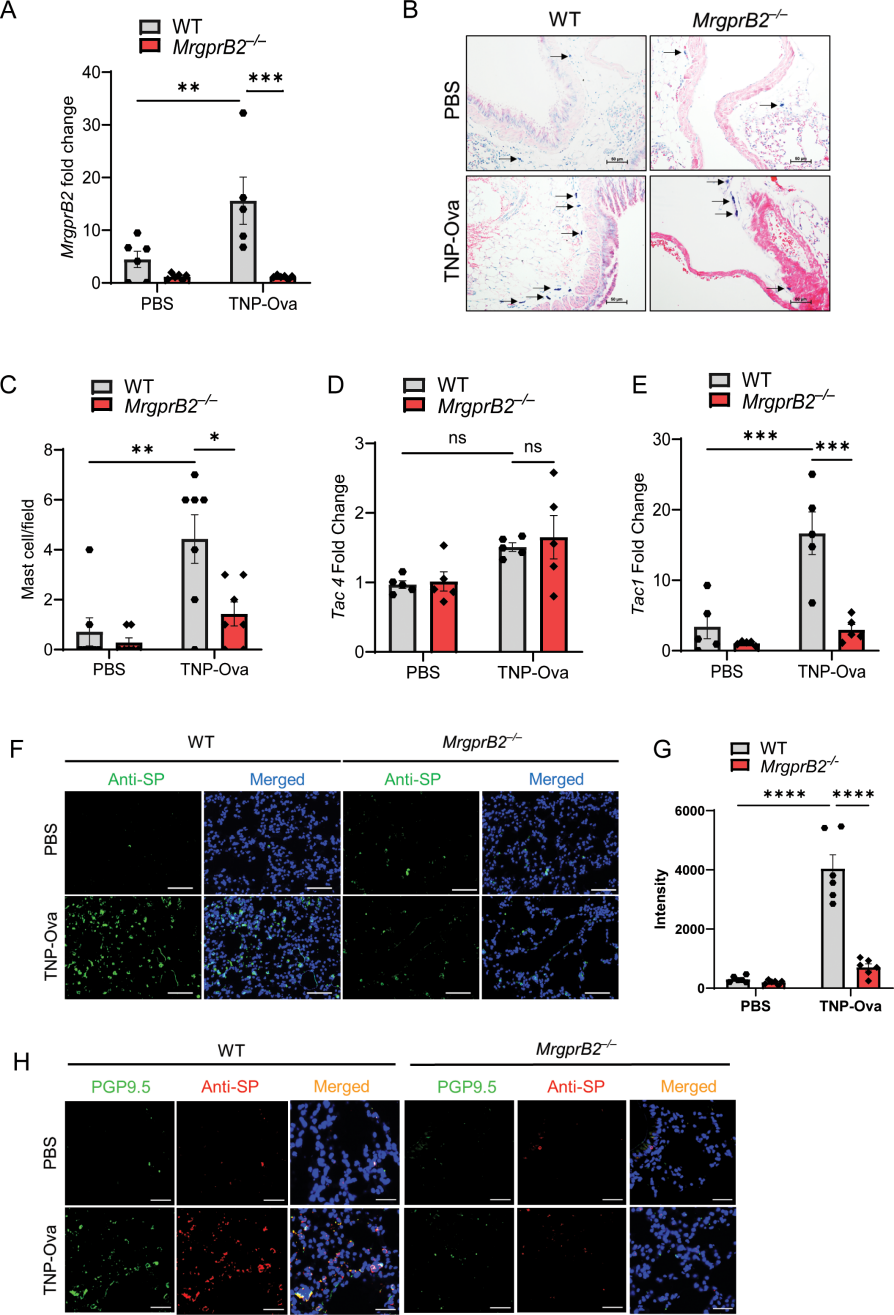
MrgprB2 contributes to MC recruitment and upregulation of SP in the lungs of IgE-sensitized and antigen-challenged mice. **(A)** Expression of MrgprB2 transcript in the lungs of WT and *MrgprB2^−/−^* experimental mice. **(B)** Representative images of toluidine blue-stained MCs in the lung sections (scale bar, 50 µm). **(C)** MC count per field (n = 6–7). mRNA expression of **(D)** *Tac4* and **(E)** *Tac1* in the lungs of WT and *MrgprB2^−/−^* mice (n = 5–6). **(F)** Representative images of immunofluorescence staining of SP expression (anti-SP; green) and nuclear counterstain DAPI (blue) in PBS and IgE-sensitized/antigen-challenged mice. **(G)** Intensity of anti-SP per field (n = 6). **(H)** Representative images of immunofluorescence staining of SP expression (anti-SP; red), PGP9.5 (green), and nuclear counterstain DAPI (blue) in PBS and IgE-sensitized and challenged mice. Data were analyzed using two-way ANOVA with Tukey’s multiple comparisons test; error bars are presented as mean ± SEM. Significant differences were set at *p < 0.05, **p < 0.01, ***p < 0.001 and ****p < 0.0001; ns, non-significant. MC, mast cell; SP, substance P; WT, wild type; PBS, phosphate-buffered saline.

Jin et al. (6) recently utilized IgE sensitization and antigen challenge to determine the role of MC-derived cytokines in AAI *in vivo*. We used a similar approach to determine if MrgprB2 contributes to IgE-mediated responses. For this, we passively sensitized WT and *MrgprB2^−/−^* mice with TNP-specific IgE, challenged with antigen (TNP-Ova), and sacrificed the mice the following day. As expected, no transcript for *MrgprB2* could be detected in the lung of *MrgprB2^−/−^* mice before or after IgE sensitization and antigen challenge (**Figure 1A**). However, *MrgprB2* transcript was present in the lung of WT mice, and this was significantly increased in the lung following IgE-sensitized and antigen challenge. To determine if this correlated with increased MC recruitment, we performed toluidine blue staining of lung sections. As shown in **Figures 1B, C**, IgE/antigen exposure resulted in a significant increase in MC numbers in the lung when compared to the control challenge. However, this increase was significantly reduced in the lung of *MrgprB2^−/−^* mice. These findings suggest that following IgE/antigen exposure, MCs are recruited to the lung through signaling via MrgprB2.

Airway sensory neurons express FcεRI, and its activation results in increased expression of *Tac1* and the release of its gene product SP, which serves to amplify type 2 (AAI) in mice (12). In addition, FcεRI stimulation of MCs promotes autocrine secretion of the neuropeptide hemokinin-1 (HK-1), which provides an adjuvant effect for the development of MC-mediated AAI (48). We therefore sought to determine if IgE/antigen exposure results in the upregulation of *Tac1* and *Tac4* transcripts (encoding SP and HK-1, respectively). As shown in **Figure 1D**, IgE/antigen exposure resulted in no significant increase in the expression of *Tac4* transcript in either WT or *MrgprB2^−/−^* mice. By contrast, there was a ~6-fold increase in *Tac1* expression in WT mice, but this response was abolished in *MrgprB2^−/−^* (**Figure 1E**). We also performed immunofluorescence staining of the lung sections to determine if the changes in *Tac1* expression correlated with the generation of SP. As shown in **Figures 1F, G**, IgE/antigen exposure resulted in increased SP expression in WT mice, but this response was substantially reduced in *MrgprB2^−/−^* mice.

Serhan et al. (5) recently showed that SP expression is restricted to PGP9.5+ mouse cutaneous neuronal fibers. An immunofluorescence study using a PGP9.5 antibody has recently been used to characterize the presence of nerve fibers in whole-mount human airway biopsies (49). Furthermore, in guinea pigs, PGP9.5+ and SP immunoreactive fibers are increased during respiratory syncytial virus infection (47). We therefore used a double immunofluorescence staining to determine the expression of PGP9.5+ nerve fibers (Green) and SP (Red) in mouse lungs following IgE sensitization and antigen challenge (**Figure 1H**). We found that compared to control, IgE/antigen caused a substantial increase in PGP9.5+ immunofluorescence, which colocalized with SP. Furthermore, this increase in double immunofluorescence was substantially reduced in *MrgprB2^−/−^* mice (**Figure 1H**).

### MrgprB2 deletion results in reduced cytokine generation, AAI, and goblet cell hyperplasia

**Figure 2:**
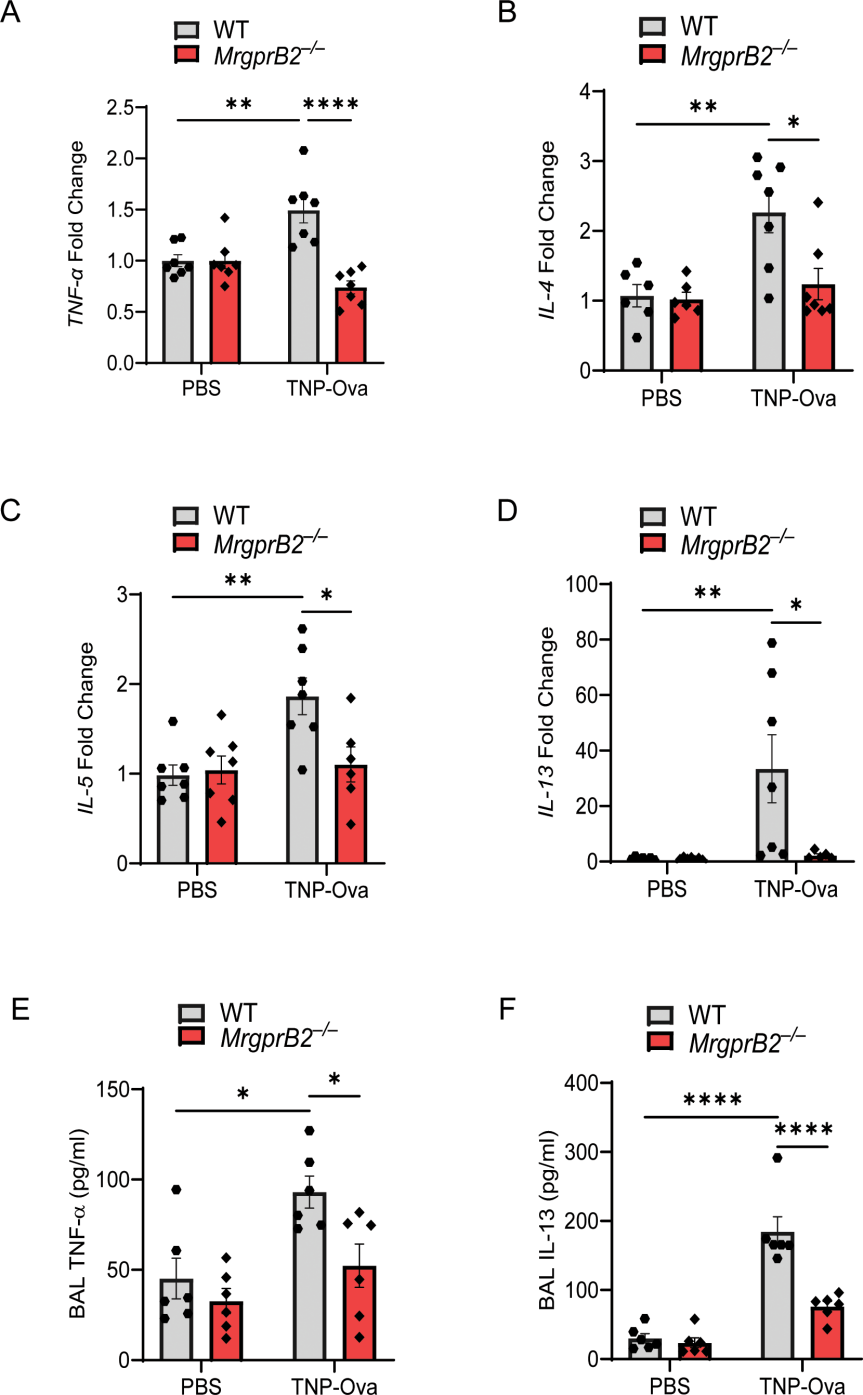
MrgprB2 deletion results in reduced cytokine generation in the lungs following IgE sensitization and antigen challenge. Transcripts for **(A)** *TNF-α*, **(B)** *IL-4*, **(C)** *IL-5*, and **(D)** *IL-13* in lung tissue following IgE sensitization/antigen challenge were assessed by RT-PCR (n = 6/group). The production of cytokine **(E)**, TNF-α, and **(F)** IL-13 from the BAL of the experimental mouse group was quantified by ELISA (n = 6–8/group). Data were analyzed using two-way ANOVA with Tukey’s multiple comparisons test; error bars are presented as mean ± SEM. Significant differences were set at *p < 0.05, **p < 0.01, and ****p < 0.0001. BAL, bronchoalveolar lavage.

MC-derived TNF-α contributes to the pathogenesis of AAI, likely via Th2 cytokine production (50). Studies with human bronchial biopsy samples from subjects with asthma showed that MCs express IL-4 and IL-13 (2). Furthermore, IL-5 contributes to eosinophil activation and bronchial hyperreactivity (51). Jin et al. (6) recently showed that aggregation of FcεRI on mouse bone marrow-derived MCs (BMMCs) results in the generation of TNF-α, IL-4, IL-5, and IL-13. Therefore, we quantitated the transcripts for cytokines in the lung of IgE-sensitized/antigen-challenged WT and *MrgprB2^−/−^* mice. As shown in **Figures 2A–D**, IgE/antigen exposure resulted in the enhancement of *TNF-α*, *IL-4*, *IL-5*, and *IL-13* transcripts, and these responses were significantly reduced in *MrgprB2^−/−^* mice. We also measured the generation of TNF-α and IL-13 in BAL. As shown in **Figures 2E, F**, IgE/antigen exposure resulted in the secretion of TNF-α and IL-13, and these responses were significantly reduced in *MrgprB2^−/−^* mice.

**Figure 3:**
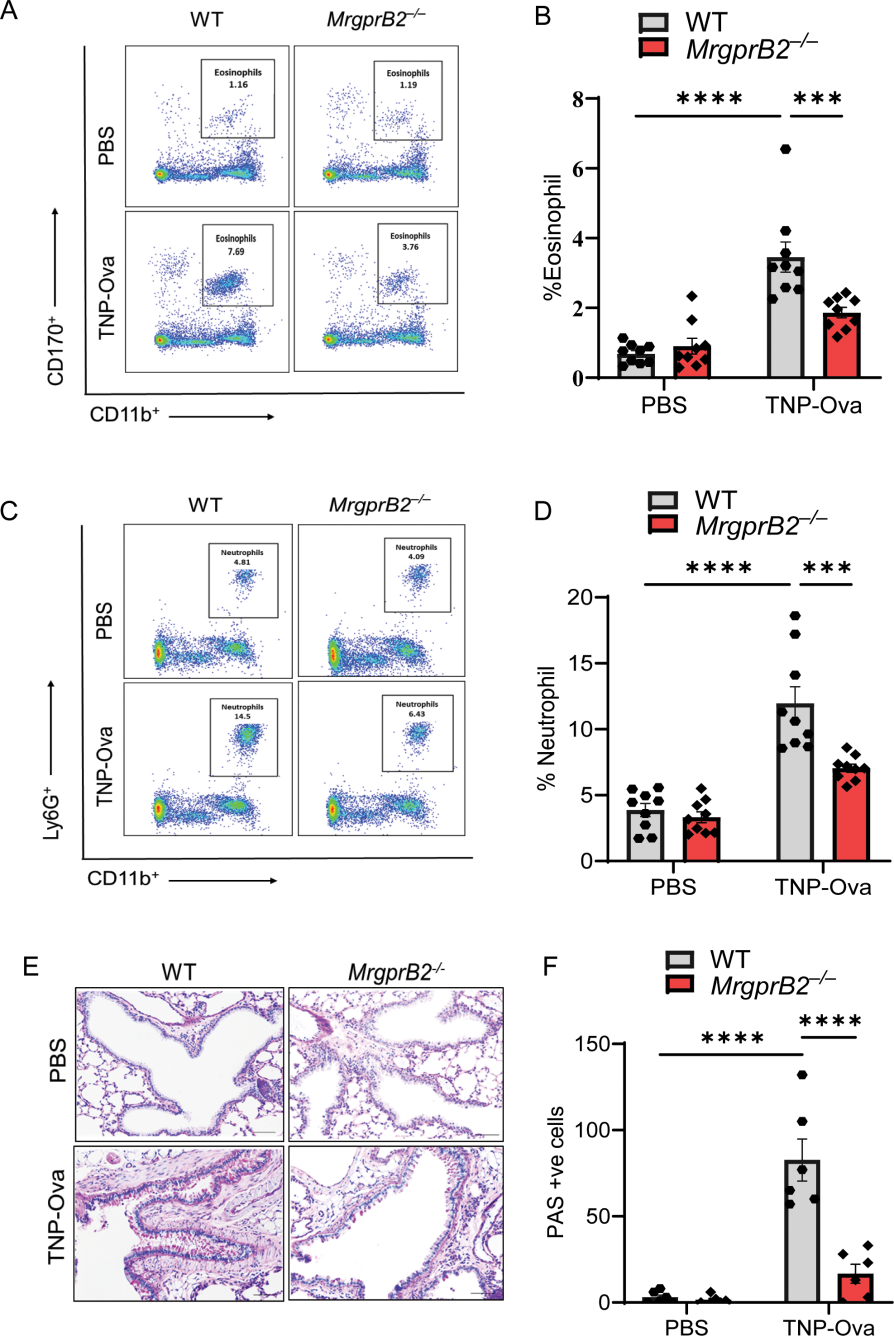
Absence of MrgprB2 results in reduced inflammatory cell infiltrate and goblet cell hyperplasia in lung tissue of IgE-sensitized and antigen-challenged mice. **(A, B)** Flow cytometry analysis of eosinophils. Cells from lung digest were stained with fluorescent anti-CD45, anti-CD11b, and anti-CD170 to analyze eosinophil subsets. Representative plots of separate experiments are presented. Percentages of CD11b^+^CD170^+^ (eosinophils) within CD45^+^ cells are shown for lungs from WT and *MrgprB2^−/−^* mice. **(C, D)** Flow cytometry analysis of neutrophils. Cells from lung digest were stained with fluorescent anti-CD45, anti-CD11b, and anti-Ly6G to analyze neutrophil subsets. Percentages of CD11b^+^Ly6G^+^ (neutrophils) within CD45^+^ cells are shown for lungs from WT and *MrgprB2^−/−^* mice of different experimental groups. **(E)** Lung tissue sections were stained with periodic acid–Schiff (PAS) for mucus secretion in the experimental groups. **(F)** Quantitative mucus-producing (PAS^+^) cells in the bronchiolar lumen. Magnification, ×20; scale bar, 50 µm. Data were analyzed using two-way ANOVA with Tukey’s multiple comparisons test; error bars are presented as mean ± SEM (n = 6–8 mice/group). Significant differences were set at ***p < 0.001 and ****p < 0.0001. WT, wild type. PBS, phosphate-buffered saline; TNP-Ova, 2,4,6-trinitrophenyl ovalbumin; PAS, periodic acid–Schiff.

To determine if the induction of these cytokines is associated with leukocyte recruitment, we first performed H&E staining of lung sections. We found an increased number of inflammatory cells around the bronchioles of WT mice following IgE/antigen exposure, but this response was almost absent in *MrgprB2^−/−^* mice (**Supplementary Figure S1A**). We then used cytospin preparations of BAL cells for immunofluorescence staining of eosinophils. As shown in **Supplementary Figures S1B, C**, IgE/antigen exposure resulted in eosinophil recruitment, which was substantially reduced in *MrgprB2^−/−^* mice. We also used lung digest for flow cytometry analysis for eosinophil and neutrophil recruitment (gating strategy, **Supplementary Figure S5**). Antigen challenge resulted in both eosinophil and neutrophil recruitment in the lung of WT mice, but these responses were significantly reduced in *MrgprB2^−/−^* mice (**Figures 3A–D**). Reduced eosinophil (**Supplementary Figure S2A**) and neutrophil (**Supplementary Figure S2B**) recruitment were also observed after immunofluorescence staining in paraffin-embedded lung tissue sections of the same experimental mice. The increase in PAS-positive cells among the bronchial epithelial cells indicates goblet cell hyperplasia (52). To detect goblet cells in the bronchiolar epithelium, lung tissue sections were stained with PAS. IgE/antigen exposure resulted in an increased number of PAS-positive cells in WT mice, but this response was significantly reduced in *MrgprB2^−/−^* mice (**Figures 3E, F**).

### β-arr2 expressed in MCs contributes to IgE-mediated MC recruitment and AAI

**Figure 4:**
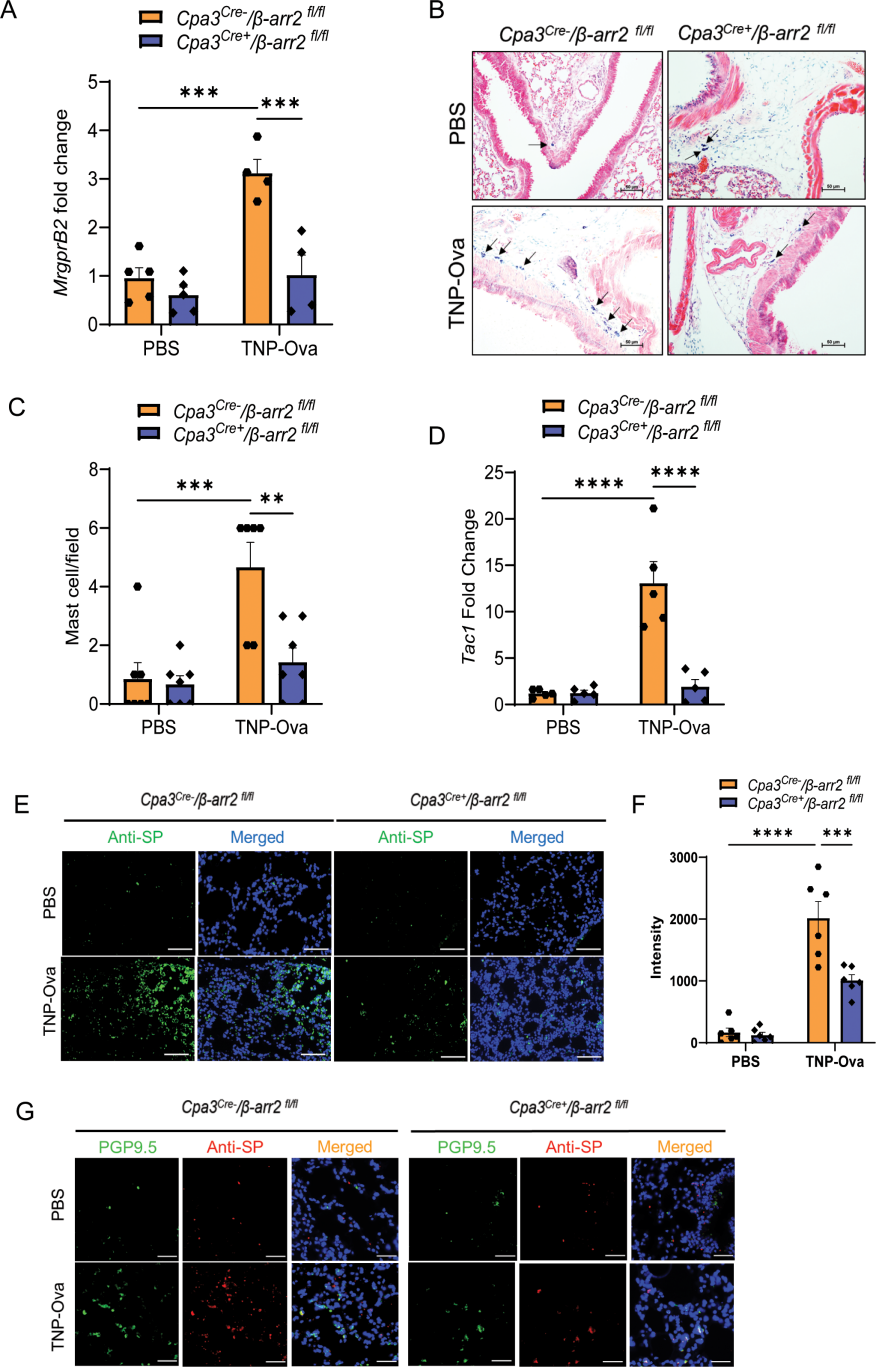
β-arr2 contributes to MC recruitment and upregulation of SP in the lungs of IgE-sensitized and antigen-challenged mice. **(A)** Expression of MrgprB2 transcript in the lungs of *Cpa3^Cre−^*/*β-arr2^fl/fl^* and *Cpa3^Cre+^*/*β-arr2^fl/fl^* experimental mice. **(B)** Representative images of toluidine blue-stained MCs in the lung sections of experimental mice (scale bar, 50 µm). **(C)** MC count per field (n = 6–7). **(D)** *Tac1* mRNA expression in the lungs of *Cpa3^Cre−^*/*β-arr2^fl/fl^* and *Cpa3^Cre+^*/*β-arr2^fl/fl^* experimental mice. **(E)** Representative image of immunofluorescence staining of SP expression (anti-SP; green) and nuclear counterstain DAPI (blue) in PBS and IgE-sensitized and challenged mice. **(F)** Intensity of anti-SP per field (n = 6). **(G)** Representative images of immunofluorescence staining of SP expression (anti-SP; red), PGP9.5 (green), and nuclear counterstain DAPI (blue). Data were analyzed using two-way ANOVA with Tukey’s multiple comparisons test; error bars are presented as mean ± SEM. Significant differences were set at **p < 0.01, ***p < 0.001, and ****p < 0.0001. MC, mast cell; SP, substance P; PBS, phosphate-buffered saline.

**Figure 5:**
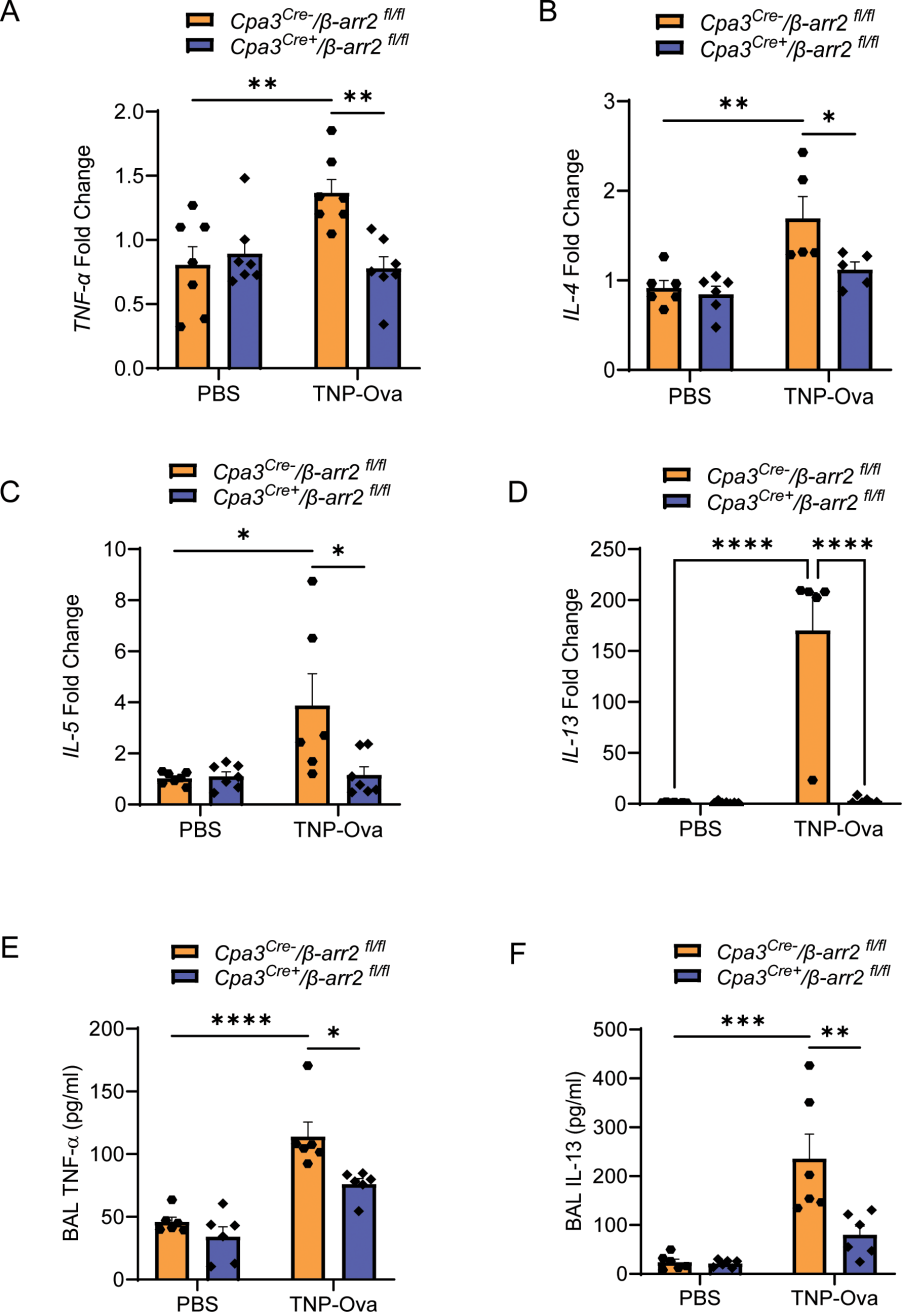
MC-specific deletion of β-arr2 results in reduced IgE-mediated cytokine generation. Expression of **(A)** *TNF-α*, **(B)** *IL-4*, **(C)** *IL-5*, and **(D)** *IL-13* transcripts were assessed by RT-PCR on isolated mRNA samples (n = 5–6/group). Each symbol represents one mouse. The production of cytokine **(E)**, TNF-α, and **(F)** IL-13 from the BAL of the experimental mouse group was quantified by ELISA. Data were analyzed using two-way ANOVA with Tukey’s multiple comparisons test; error bars are presented as mean ± SEM. Significant differences were set at *p < 0.05, **p < 0.01, ***p < 0.001, and ****p < 0.0001. MC, mast cell; BAL, bronchoalveolar lavage.

β-arr2 expressed in hematopoietic cells is required for eosinophil recruitment in the development of AAI in mice through dephosphorylation of the actin-binding protein cofilin (36, 38). We have recently shown that MrgprB2-mediated β-arr2 biased signaling for MC chemotaxis through dephosphorylation of cofilin contributes to rosacea-like inflammation in mice (39). Furthermore, the findings described above that decreased MC number in *MrgprB2^−/−^* mice is associated with reduced cytokine generation, AAI, and mucus secretion raises the interesting possibility that β-arr2-mediated MC recruitment is required to induce these responses. To test this possibility, we utilized *Cpa3^Cre+^*/*β-arr2^fl/fl^* and *Cpa3^Cre−^*/*β-arr2^fl/fl^* mice for IgE sensitization and challenge studies as described for *MrgprB2^−/−^* mice above. We found that MC-specific deletion of β-arr2 resulted in almost complete inhibition of expression of *MrgprB2* transcript (**Figure 4A**), lung MC count (**Figures 4B, C**), *Tac1* transcript (**Figure 4D**), and SP generation (**Figures 4E, F**) and its localization with PGP9.5 (**Figure 4G**). The absence of β-arr2 in MCs also resulted in a significant reduction of *TNF-α*, *IL-4*, *IL-5*, and *IL-13* transcripts in lung tissue (**Figures 5A–D**) and cytokines in BAL (**Figures 5E, F**).

**Figure 6:**
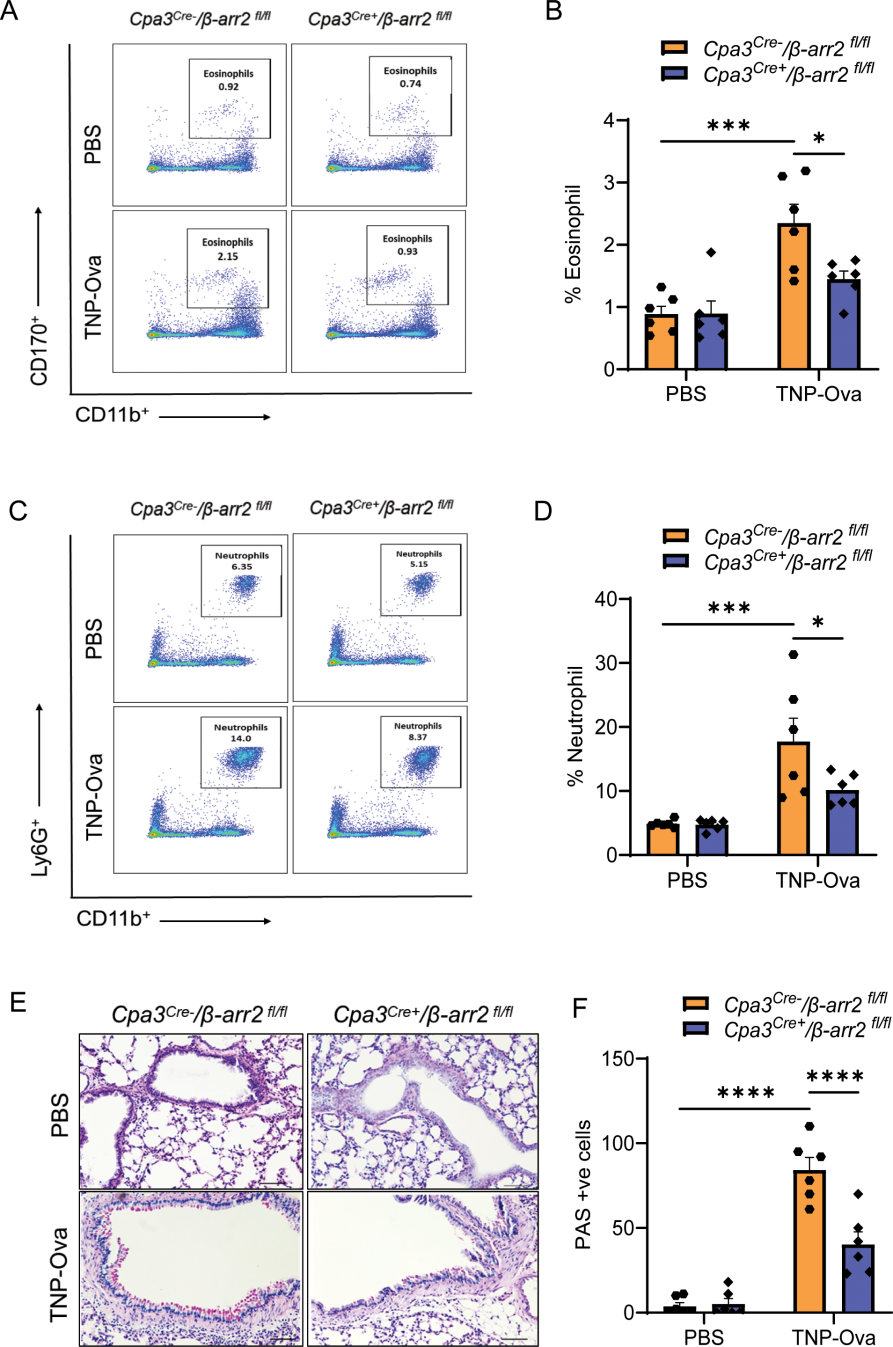
Reduced inflammatory cell infiltrate and goblet cell hyperplasia in lung tissue of mice with MC-specific deletion of β-arr2. **(A, B)** Flow cytometry analysis of eosinophils. Cells from lung digest were stained with fluorescent anti-CD45, anti-CD11b, and anti-CD170 to analyze eosinophil subsets. Representative plots of separate experiments are presented. Percentages of CD11b^+^CD170^+^ (eosinophils) within CD45^+^ cells are shown for lungs from *Cpa3^Cre−^*/*β-arr2^fl/fl^* and *Cpa3^Cre+^*/*β-arr2^fl/fl^* mice of different experimental groups. **(C, D)** Flow cytometry analysis of neutrophils. Cells from lung digest were stained with fluorescent anti-CD45, anti-CD11b, and anti-Ly6G to analyze neutrophil subsets by flow cytometry. Percentages of CD11b^+^Ly6G^+^ (neutrophils) within CD45^+^ cells are shown for lungs from *Cpa3^Cre−^*/*β-arr2^fl/fl^* and *Cpa3^Cre+^*/*β-arr2^fl/fl^* mice treated with PBS and TNP-Ova. **(E)** Lung tissue sections were stained with PAS for mucus secretion in the experimental groups. **(F)** Quantitative mucus-producing (PAS^+^) cells in the bronchiolar lumen. Magnification, ×20; scale bar, 50 µm. Data were analyzed using two-way ANOVA with Tukey’s multiple comparisons test; error bars are presented as mean ± SEM (n = 6–8 mice/group). Significant differences were set at *p < 0.05, ***p < 0.001, and ****p < 0.0001. MC, mast cell; PBS, phosphate-buffered saline; TNP-Ova, 2,4,6-trinitrophenyl ovalbumin; PAS, periodic acid–Schiff.

IgE/antigen exposure caused inflammatory cell recruitment around the bronchioles of control (*Cpa3^Cre−^*/*β-arr2^fl/fl^*) mice, but this was substantially reduced in *Cpa3^Cre+^*/*β-arr2^fl/fl^* mice (**Supplementary Figure S3A**). The increased number of eosinophils in BAL (**Supplementary Figure S3B, C**) was also significantly reduced in *Cpa3^Cre+^*/*β-arr2^fl/fl^* mice. Similarly, neutrophil and eosinophil recruitment in lung digest and PAS staining in the lung tissue, following IgE/antigen challenge, were significantly reduced in the absence of β-arr2 in MCs (**Figures 6A–F**). Reduced eosinophil (**Supplementary Figure S4A**) and neutrophil (**Supplementary Figure S4B**) recruitment were also observed after immunofluorescence staining in paraffin-embedded lung tissue sections of the same experimental mice. These findings suggest that IgE-mediated cytokine generation and AAI require β-arr2-mediated MC recruitment.

## Discussion

Infiltration of the airway by MCs and the generation of TNF-α and the Th2 cytokines IL-4, IL-5, and IL-13 (2, 50) contribute to disordered airway function found in asthma (1). Furthermore, the level of SP is elevated in lungs, BAL, and sputum obtained from asthmatic patients compared to normal controls, and these MCs undergo degranulation in response to SP (18–20). These findings suggest that neuroimmune interaction involving SP and MCs contributes to asthma, but the mechanism of this crosstalk has not been determined. Type 2 immune response in AD involves MrgprB2 activation in MCs by SP, and we have recently shown an increased level of MRGPRX2-expressing MCs is associated with severe asthma (5, 24). Based on these findings, we hypothesized that SP contributes to allergic asthma by activating MCs via MRGPRX2. Using *MrgprB2^−/−^* mice, we demonstrated that Th2 cytokine generation, AAI, and mucus secretion are associated with the generation of SP from nerve endings and activation of MCs via MrgprB2. We also propose that β-arr2 contributes to MrgprB2-mediated MC recruitment to facilitate eosinophilic and neutrophilic inflammation in AAI.

While MCs are known to orchestrate the development of allergic asthma in humans, many of the *in vivo* models in mice induce AAI without involving MCs. The best-characterized model for the study of MC-dependent model AAI involves ovalbumin sensitization and challenge in the absence of adjuvant, and this is associated with the release of TNF-α and Th2 cytokines (IL-4, IL-5, and IL-13) from MCs, resulting in neutrophil and eosinophil infiltration of the lungs (48, 50). Using this model, Sumpter et al. (48) showed that FcϵRI activation of MCs promotes autocrine expression of *Tac4* and secretion of its gene product HK-1, which signals through neurokinin-1 receptor (NK-1R) to provide adjuvancy for efficient AAI. However, we were unable to demonstrate an increase in *Tac4*, and this could reflect differences in the method of sensitization and challenge used. Our approach in this study was to use passive IgE sensitization and antigen challenge instead of active sensitization for two reasons. First, it provides a rapid and previously documented procedure for inducing AAI by targeting the FcεRI in MCs (6). Second, it has been shown that vagal nociceptor neurons express FcϵRI, and activation of this receptor results in *Tac1* expression and release of SP that helps to amplify AAI. Using this procedure, we found that the *Tac1* expression and SP generation from nerve endings are induced in WT mice, which is associated with the upregulation of TNF-α, Th2 cytokines, AAI, and mucus secretion. However, these responses are substantially reduced in *MrgprB2^−/−^* mice. These findings suggest that SP released from nociceptors following FcϵRI activates MrgprB2 on MCs to facilitate AAI in mice. Thus, AD, neurogenic inflammation, and AAI share common features regarding neuroimmune interaction in which the release of SP from nociceptors activates MCs via MrgprB2 to contribute to their pathogenesis (5, 10). Given that MRGPRX2 is the human counterpart of mouse MrgprB2, targeting this receptor may provide novel approaches for modulating AD, neurogenic inflammation, and AAI.

An interesting finding of the present study was that the absence of MrgprB2 resulted in substantial inhibition of *Tac1* expression and SP generation in the lungs of IgE-sensitized/antigen-challenged mice. If SP is produced from nociceptors to activate lung MCs via MrgprB2, then it is not clear how this GPCR contributes to *Tac1* expression and SP generation. It is well documented that in human asthma, MCs infiltrate into the lung, and this is associated with lung MC chemotaxis *in vitro* (1, 53–55). We found that the absence of MrgprB2 resulted in decreased MC recruitment in mouse lungs, and this was associated with attenuation of TNF-α, Th2 cytokine generation, AAI, and goblet cell hyperplasia. Previous studies have shown that these cytokines are generated following FcεRI aggregation of mouse MCs (6, 48). These findings suggest that MrgprB2-mediated MC recruitment facilitates bidirectional interaction between FcεRI in nociceptors and MCs to promote cytokine generation and AAI.

The mechanism via which MrgprB2 contributes to MC recruitment in AAI is not known. However, we have recently shown that β-arr2 expressed in MCs contributes to the development of rosacea-like inflammation in mice by promoting MC chemotaxis through dephosphorylation of cofilin (26). In addition, chemokine- and protease-induced Chemotaxis of Th2 cells and eosinophils are also mediated via β-arr2 mediated cofilin dephosphorylation signaling pathway (26, 34, 35). β-arr2 expressed in hematopoietic cells and structural cells promotes Th2 cell/eosinophil recruitment and causes airway hyperresponsiveness, respectively, in a mouse model of asthma (38). However, the demonstration in the present study that loss of either MrgprB2 or β-arr2 in MCs results in decreased MC recruitment, cytokine generation, and AAI suggests that β-arr2-biased signaling via MrgprB2 contributes to the pathogenesis of allergic asthma and provides a novel target for modulating experimental disease phenotype in mice.

Before the discovery of MrgprB2, neuropeptides such as SP were thought to activate MCs via the neurokinin receptor NK-1R (48). However, NK-1R antagonists that modulate inflammation in mice have no effect in humans, emphasizing the challenge of translating findings from animal models to the clinic (56). More recently, however, it was shown that conventional NK-1R antagonists have off-target activity on MrgprB2, but not its human counterpart MRGPRX2 (56). By contrast, a small molecule MRGPRX2 inhibitor blocks degranulation in response to a variety of receptor agonists in human MCs but has no effect in mouse MCs (57). This difference likely reflects ~53% sequence homology between MRGPRX2 and MrgprB2. Given our previous demonstration that MRGPRX2-expressing MCs are increased in lung MCs of asthmatics (24) and the finding in the present study that MrgprB2-mediated β-arr2 biased signaling contributes to AAI suggests that specific MRGPRX2 antagonists could be utilized for the treatment of allergic asthma in humans. In addition to AAI, MrgprB2 expressed in MCs also contributes to AD, systemic anaphylaxis, and peanut allergy (8, 58). Thus, receptor could be targeted for the modulation of atopic reactions that are generally thought to be IgE/FcεRI mediated.

## Data Availability

The original contributions presented in the study are included in the article/**Supplementary Material**. Further inquiries can be directed to the corresponding author.

## References

[1] Brightling CE Bradding P Symon FA Holgate ST Wardlaw AJ Pavord ID. Mast-cell infiltration of airway smooth muscle in asthma. N Engl J Med. (2002) 346:1699–705. doi: 10.1056/NEJMoa012705 12037149

[2] Brightling CE Symon FA Holgate ST Wardlaw AJ Pavord ID Bradding P. Interleukin-4 and -13 expression is co-localized to mast cells within the airway smooth muscle in asthma. Clin Exp Allergy. (2003) 33:1711–6. doi: 10.1111/j.1365-2222.2003.01827.x 14656359

[3] Page S Ammit AJ Black JL Armour CL. Human mast cell and airway smooth muscle cell interactions: implications for asthma. Am J Physiol Lung Cell Mol Physiol. (2001) 281:L1313–23. doi: 10.1152/ajplung.2001.281.6.L1313 11704524

[4] Robinson DS. The role of the mast cell in asthma: induction of airway hyperresponsiveness by interaction with smooth muscle? J Allergy Clin Immunol. (2004) 114:58–65. doi: 10.1016/j.jaci.2004.03.034 15241345

[5] Serhan N Basso L Sibilano R Petitfils C Meixiong J Bonnart C. House dust mites activate nociceptor-mast cell clusters to drive type 2 skin inflammation. Nat Immunol. (2019) 20:1435–43. doi: 10.1038/s41590-019-0493-z PMC6858877 31591569

[6] Jin C Shelburne CP Li G Potts EN Riebe KJ Sempowski GD. Particulate allergens potentiate allergic asthma in mice through sustained IgE-mediated mast cell activation. J Clin Invest. (2011) 121:941–55. doi: 10.1172/JCI43584 PMC3049384 21285515

[7] McNeil BD Pundir P Meeker S Han L Undem BJ Kulka M. Identification of a mast-cell-specific receptor crucial for pseudo-allergic drug reactions. Nature. (2015) 519:237–41. doi: 10.1038/nature14022 PMC4359082 25517090

[8] Tauber M Basso L Martin J Bostan L Pinto MM Thierry GR. Landscape of mast cell populations across organs in mice and humans. J Exp Med. (2023) 220(10):e20230570. doi: 10.1084/jem.20230570 37462672 PMC10354537

[9] Stead RH Tomioka M Quinonez G Simon GT Felten SY Bienenstock J. Intestinal mucosal mast cells in normal and nematode-infected rat intestines are in intimate contact with peptidergic nerves. Proc Natl Acad Sci U S A. (1987) 84:2975–9. doi: 10.1073/pnas.84.9.2975 PMC304783 2437589

[10] Green DP Limjunyawong N Gour N Pundir P Dong X. A mast-cell-specific receptor mediates neurogenic inflammation and pain. Neuron. (2019) 101:412–20 e3. doi: 10.1016/j.neuron.2019.01.012 30686732 PMC6462816

[11] Van Remoortel S Lambeets L De Winter B Dong X Rodriguez Ruiz JP Kumar-Singh S. Mrgprb2-dependent mast cell activation plays a crucial role in acute colitis. Cell Mol Gastroenterol Hepatol. (2024) 18(5):101391. doi: 10.1016/j.jcmgh.2024.101391 39179175 PMC11462171

[12] Crosson T Wang JC Doyle B Merrison H Balood M Parrin A. FcepsilonR1-expressing nociceptors trigger allergic airway inflammation. J Allergy Clin Immunol. (2021) 147:2330–42. doi: 10.1016/j.jaci.2020.12.644 PMC9004488 33453289

[13] Irani AA Schechter NM Craig SS DeBlois G Schwartz LB. Two types of human mast cells that have distinct neutral protease compositions. Proc Natl Acad Sci U S A. (1986) 83:4464–8. doi: 10.1073/pnas.83.12.4464 PMC323754 3520574

[14] Oskeritzian CA Zhao W Min HK Xia HZ Pozez A Kiev J. Surface CD88 functionally distinguishes the MCTC from the MCT type of human lung mast cell. J Allergy Clin Immunol. (2005) 115:1162–8. doi: 10.1016/j.jaci.2005.02.022 PMC1460014 15940129

[15] Dwyer DF Ordovas-Montanes J Allon SJ Buchheit KM Vukovic M Derakhshan T. Human airway mast cells proliferate and acquire distinct inflammation-driven phenotypes during type 2 inflammation. Sci Immunol. (2021) 6(56):eabb7221. doi: 10.1126/sciimmunol.abb7221 33637594 PMC8362933

[16] Fujisawa D Kashiwakura J Kita H Kikukawa Y Fujitani Y Sasaki-Sakamoto T. Expression of Mas-related gene X2 on mast cells is upregulated in the skin of patients with severe chronic urticaria. J Allergy Clin Immunol. (2014) 134:622–33 e9. doi: 10.1016/j.jaci.2014.05.004 24954276

[17] Plum T Wang X Rettel M Krijgsveld J Feyerabend TB Rodewald HR. Human mast cell proteome reveals unique lineage, putative functions, and structural basis for cell ablation. Immunity. (2020) 52:404–16 e5. doi: 10.1016/j.immuni.2020.01.012 32049054

[18] Tomaki M Ichinose M Miura M Hirayama Y Yamauchi H Nakajima N. Elevated substance P content in induced sputum from patients with asthma and patients with chronic bronchitis. Am J Respir Crit Care Med. (1995) 151:613–7. doi: 10.1164/ajrccm/151.3_Pt_1.613 7533601

[19] Nieber K Baumgarten CR Rathsack R Furkert J Oehme P Kunkel G. Substance P and beta-endorphin-like immunoreactivity in lavage fluids of subjects with and without allergic asthma. J Allergy Clin Immunol. (1992) 90:646–52. doi: 10.1016/0091-6749(92)90138-R 1383307

[20] Heaney LG Cross LJ Stanford CF Ennis M. Substance P induces histamine release from human pulmonary mast cells. Clin Exp Allergy. (1995) 25:179–86. doi: 10.1111/j.1365-2222.1995.tb01024.x 7538443

[21] Andersson CK Bergqvist A Mori M Mauad T Bjermer L Erjefalt JS. Mast cell-associated alveolar inflammation in patients with atopic uncontrolled asthma. J Allergy Clin Immunol. (2011) 127:905–12 e1-7. doi: 10.1016/j.jaci.2011.01.022 21388666

[22] Balzar S Chu HW Strand M Wenzel S. Relationship of small airway chymase-positive mast cells and lung function in severe asthma. Am J Respir Crit Care Med. (2005) 171:431–9. doi: 10.1164/rccm.200407-949OC 15563633

[23] Sverrild A Bergqvist A Baines KJ Porsbjerg C Andersson CK Thomsen SF. Airway responsiveness to mannitol in asthma is associated with chymase-positive mast cells and eosinophilic airway inflammation. Clin Exp Allergy. (2016) 46:288–97. doi: 10.1111/cea.2016.46.issue-2 26252943

[24] Manorak W Idahosa C Gupta K Roy S Panettieri R Jr. Ali H. Upregulation of Mas-related G Protein coupled receptor X2 in asthmatic lung mast cells and its activation by the novel neuropeptide hemokinin-1. Respir Res. (2018) 19:1. doi: 10.1186/s12931-017-0698-3 29295703 PMC5751818

[25] Vijayanand P Durkin K Hartmann G Morjaria J Seumois G Staples KJ. Chemokine receptor 4 plays a key role in T cell recruitment into the airways of asthmatic patients. J Immunol. (2010) 184:4568–74. doi: 10.4049/jimmunol.0901342 20237293

[26] DeFea K. Arresting CCR4: A new look at an old approach to combating asthma. Am J Respir Cell Mol Biol. (2018) 58:673–5. doi: 10.1165/rcmb.2017-0396ED 29856260

[27] Schiff HV Rivas CM Pederson WP Sandoval E Gillman S Prisco J. beta-Arrestin-biased proteinase-activated receptor-2 antagonist C781 limits allergen-induced airway hyperresponsiveness and inflammation. Br J Pharmacol. (2023) 180:667–80. doi: 10.1111/bph.v180.5 PMC10311467 35735078

[28] Lefkowitz RJ Whalen EJ. beta-arrestins: traffic cops of cell signaling. Curr Opin Cell Biol. (2004) 16:162–8. doi: 10.1016/j.ceb.2004.01.001 15196559

[29] Cahill TJ 3rd Thomsen AR Tarrasch JT Plouffe B Nguyen AH Yang F. Distinct conformations of GPCR-beta-arrestin complexes mediate desensitization, signaling, and endocytosis. Proc Natl Acad Sci U S A. (2017) 114:2562–7. doi: 10.1073/pnas.1701529114 PMC5347553 28223524

[30] Lefkowitz RJ. Arrestins come of age: a personal historical perspective. Prog Mol Biol Transl Sci. (2013) 118:3–18. doi: 10.1016/B978-0-12-394440-5.00001-2 23764048

[31] Ahn S Shenoy SK Luttrell LM Lefkowitz RJ. SnapShot: beta-arrestin functions. Cell. (2020) 182:1362– e1. doi: 10.1016/j.cell.2020.07.034 32888497

[32] Zoudilova M Min J Richards HL Carter D Huang T DeFea KA. beta-Arrestins scaffold cofilin with chronophin to direct localized actin filament severing and membrane protrusions downstream of protease-activated receptor-2. J Biol Chem. (2010) 285:14318–29. doi: 10.1074/jbc.M109.055806 PMC2863192 20207744

[33] McGovern KW DeFea KA. Molecular mechanisms underlying beta-arrestin-dependent chemotaxis and actin-cytoskeletal reorganization. Handb Exp Pharmacol. (2014) 219:341–59. doi: 10.1007/978-3-642-41199-1_17 24292838

[34] Fong AM Premont RT Richardson RM Yu YR Lefkowitz RJ Patel DD. Defective lymphocyte chemotaxis in beta-arrestin2- and GRK6-deficient mice. Proc Natl Acad Sci U S A. (2002) 99:7478–83. doi: 10.1073/pnas.112198299 PMC124256 12032308

[35] Lin R Choi YH Zidar DA Walker JKL. beta-arrestin-2-dependent signaling promotes CCR4-mediated chemotaxis of murine T-helper type 2 cells. Am J Respir Cell Mol Biol. (2018) 58:745–55. doi: 10.1165/rcmb.2017-0240OC PMC6002661 29361236

[36] Yee MC Nichols HL Polley D Saifeddine M Pal K Lee K. Protease-activated receptor-2 signaling through beta-arrestin-2 mediates Alternaria alkaline serine protease-induced airway inflammation. Am J Physiol Lung Cell Mol Physiol. (2018) 315:L1042–L57. doi: 10.1152/ajplung.00196.2018 PMC6337008 30335499

[37] Walker JK Fong AM Lawson BL Savov JD Patel DD Schwartz DA. Beta-arrestin-2 regulates the development of allergic asthma. J Clin Invest. (2003) 112:566–74. doi: 10.1172/JCI200317265 PMC171386 12925697

[38] Hollingsworth JW Theriot BS Li Z Lawson BL Sunday M Schwartz DA. Both hematopoietic-derived and non-hematopoietic-derived β-arrestin-2 regulates murine allergic airway disease. Am J Respir Cell Mol Biol. (2010) 43:269–75. doi: 10.1165/rcmb.2009-0198OC PMC2933545 19805483

[39] Roy S Alkanfari I Chaki S Ali H. Role of mrgprB2 in rosacea-like inflammation in mice: modulation by beta-arrestin 2. J Invest Dermatol. (2022) 142:2988–97 e3. doi: 10.1016/j.jid.2022.05.005 35644498 PMC9634617

[40] Alkanfari I Freeman KB Roy S Jahan T Scott RW Ali H. Small-molecule host-defense peptide mimetic antibacterial and antifungal agents activate human and mouse mast cells via mas-related GPCRs. Cells. (2019) 8(4):311. doi: 10.3390/cells8040311 30987258 PMC6523814

[41] Roy S Gupta K Ganguly A Ali H. [amp]]beta;-Arrestin2 expressed in mast cells regulates ciprofloxacin-induced pseudoallergy and IgE-mediated anaphylaxis. J Allergy Clin Immunol. (2019) 144:603–6. doi: 10.1016/j.jaci.2019.04.024 PMC6688937 31077686

[42] Li S Aliyeva M Daphtary N Martin RA Poynter ME Kostin SF. Antigen-induced mast cell expansion and bronchoconstriction in a mouse model of asthma. Am J Physiol Lung Cell Mol Physiol. (2014) 306:L196–206. doi: 10.1152/ajplung.00055.2013 PMC3920205 24285269

[43] Singer BD Mock JR D’Alessio FR Aggarwal NR Mandke P Johnston L. Flow-cytometric method for simultaneous analysis of mouse lung epithelial, endothelial, and hematopoietic lineage cells. Am J Physiol Lung Cell Mol Physiol. (2016) 310:L796–801. doi: 10.1152/ajplung.00334.2015 PMC4867353 26944088

[44] Nazaroff CD LeSuer WE Masuda MY Pyon G Lacy P Jacobsen EA. Assessment of lung eosinophils *in situ* using immunohistological staining. Methods Mol Biol. (2021) 2223:237–66. doi: 10.1007/978-1-0716-1001-5_17 PMC7869952 33226599

[45] Tokuhiro T Ishikawa A Sato H Takita S Yoshikawa A Anzai R. Oxidized phospholipids and neutrophil elastase coordinately play critical roles in NET formation. Front Cell Dev Biol. (2021) 9:718586. doi: 10.3389/fcell.2021.718586 34568331 PMC8458647

[46] Pincus AB Huang SJ Lebold KM de la Torre U Proskocil BJ Drake MG. Multicolor labeling of airway neurons and analysis of parasympathetic heterogeneity. Sci Rep. (2022) 12:5006. doi: 10.1038/s41598-022-08655-6 35322058 PMC8943012

[47] Tan YR Yang T Liu SP Xiang Y Qu F Liu HJ. Pulmonary peptidergic innervation remodeling and development of airway hyperresponsiveness induced by RSV persistent infection. Peptides. (2008) 29:47–56. doi: 10.1016/j.peptides.2007.10.020 18055066

[48] Sumpter TL Ho CH Pleet AR Tkacheva OA Shufesky WJ Rojas-Canales DM. Autocrine hemokinin-1 functions as an endogenous adjuvant for IgE-mediated mast cell inflammatory responses. J Allergy Clin Immunol. (2015) 135:1019–30 e8. doi: 10.1016/j.jaci.2014.07.036 25201259 PMC4362795

[49] West PW Canning BJ Merlo-Pich E Woodcock AA Smith JA. Morphologic characterization of nerves in whole-mount airway biopsies. Am J Respir Crit Care Med. (2015) 192:30–9. doi: 10.1164/rccm.201412-2293OC PMC4511424 25906337

[50] Nakae S Ho LH Yu M Monteforte R Iikura M Suto H. Mast cell-derived TNF contributes to airway hyperreactivity, inflammation, and TH2 cytokine production in an asthma model in mice. J Allergy Clin Immunol. (2007) 120:48–55. doi: 10.1016/j.jaci.2007.02.046 17482668

[51] Mould AW Ramsay AJ Matthaei KI Young IG Rothenberg ME Foster PS. The effect of IL-5 and eotaxin expression in the lung on eosinophil trafficking and degranulation and the induction of bronchial hyperreactivity. J Immunol. (2000) 164:2142–50. doi: 10.4049/jimmunol.164.4.2142 10657668

[52] Ge Y Cheng R Sun S Zhang S Li L Jiang J. Fangxiao Formula alleviates airway inflammation and remodeling in rats with asthma via suppression of transforming growth factor-beta/Smad3 signaling pathway. BioMed Pharmacother. (2019) 119:109429. doi: 10.1016/j.biopha.2019.109429 31505422

[53] Dahlin JS Maurer M Metcalfe DD Pejler G Sagi-Eisenberg R Nilsson G. The ingenious mast cell: Contemporary insights into mast cell behavior and function. Allergy. (2022) 77:83–99. doi: 10.1111/all.14881 33955017

[54] Brightling CE Ammit AJ Kaur D Black JL Wardlaw AJ Hughes JM. The CXCL10/CXCR3 axis mediates human lung mast cell migration to asthmatic airway smooth muscle. Am J Respir Crit Care Med. (2005) 171:1103–8. doi: 10.1164/rccm.200409-1220OC 15879427

[55] Brightling CE Bradding P. The re-emergence of the mast cell as a pivotal cell in asthma pathogenesis. Curr Allergy Asthma Rep. (2005) 5:130–5. doi: 10.1007/s11882-005-0086-9 15683613

[56] Azimi E Reddy VB Shade KC Anthony RM Talbot S Pereira PJ. Dual action of neurokinin-1 antagonists on Mas-related GPCRs. JCI Insight. (2016) 1:e89362. doi: 10.1172/jci.insight.89362 27734033 PMC5053144

[57] Bawazir M Amponnawarat A Hui Y Oskeritzian CA Ali H. Inhibition of MRGPRX2 but not FcepsilonRI or MrgprB2-mediated mast cell degranulation by a small molecule inverse receptor agonist. Front Immunol. (2022) 13:1033794. doi: 10.3389/fimmu.2022.1033794 36275683 PMC9582160

[58] Thapaliya M Ali H. GRK2 differentially regulates FcepsilonRI and MRGPRB2-mediated responses in mast cells. Front Immunol. (2023) 14:1155777. doi: 10.3389/fimmu.2023.1155777 37063868 PMC10090543

