# Supplementary material for: Mast cell MrgprB2 in neuroimmune interaction in IgE-mediated airway inflammation and its modulation by β-arrestin2
Source: Front Immunol. 2024 Oct 17;15:1470016. doi: 10.3389/fimmu.2024.1470016 (PMC11524863; doi:10.3389/fimmu.2024.1470016)
Supplement: Supplementary file 1 [file DataSheet1.docx]

**Supporting information**

**Mast cell MrgprB2 in neuroimmune interaction in IgE-mediated Type-2 airway inflammation and its modulation by β-arrestin 2**

**Sangita Sutradhar and Hydar Ali.**

**Supplementary method**

**Immunofluorescence staining for broncho-alveolar lavage (BAL) cells**

EPx staining was done following the protocol described by previously.^1^ Briefly, broncho-alveolar lavage (BAL) cells were resuspended in cold 5% BSA/PBS and were cytospinned at slow acceleration at RT. The cells were immediately fixed in 4% formaldehyde at RT. Slides were washed and incubated with permeabilization buffer (PBS containing 0.1% Tween20) and then blocked with rodent block M for half an hour at RT. Cells were then incubated with mouse anti-EPx (1:50) primary antibody overnight at 4°C. Slides were washed three times with PBS and incubated with Alexa Fluor 647‒conjugated donkey anti-mouse IgG secondary antibodies (1:750) in dark. The cells were washed and then mounted with DAPI.

**Supplementary Figure legend**

**Figure S1. MrgprB2 deletion results in reduced inflammatory cell infiltrate in the lung of IgE sensitized and antigen challenged mice.**

**(A)** Representative H&E staining showing inflammatory cell infiltrate around the bronchiolar region of experimental mice lung (scale bar; 50 µm). (**B)** Cytospins of BAL cells showing immunofluorescence staining of eosinophil peroxidase, (anti-Epx; red). Representative images are shown at 50X magnification, scale bar; 50 μm. (**C**) Quantitation of anti-EPx BAL cells. Data were analyzed using two-way ANOVA with Tukey's multiple comparisons test, error bars are presented as mean ± SEM (n = 6 mice/group). Significant differences were set at **** *p* <0.0001.

**Figure S2. MrgprB2 deletion results in reduced leukocyte recruitment in the lung of IgE sensitized and antigen challenged mice.**

(**A**) Immunofluorescence staining for eosinophil recruitment by eosinophil peroxidase, (anti-Epx); red) and (**B**) neutrophil recruitment by myeloperoxidase (anti-MPO; red) and nuclear counterstain DAPI (blue) in the lung of experimental group of mice. Representative images are shown at 20X magnification, scale bar; 100 μm.

**Figure S3. MC specific deletion of β-arr2 leads to reduced inflammatory cell infiltrate in the lung of IgE sensitized and antigen challenged mice.**

**(A)** Representative H&E staining showing inflammatory cell infiltration around the bronchiolar region of experimental mice (scale bar; 50 µm). (**B)** Cytospins of BAL cells showing immunofluorescence staining of eosinophil peroxidase, (Anti-Epx; red). Representative images are shown at 50X magnification scale bar; 50 μm. (**C**) Quantitation of anti-EPx BAL cells. Data were analyzed using two-way ANOVA with Tukey's multiple comparisons test, error bars are presented as mean ± SEM (n= 6-mice/group). Significant differences were set at **** *p* <0.0001.

**Figure S4. MC specific deletion of β-arr2 results in reduced leukocyte recruitment in the lung of IgE sensitized and antigen challenged mice.**

(**A**) Immunofluorescence staining for eosinophil recruitment by eosinophil peroxidase, (anti-Epx; red) and (**B**) neutrophil recruitment by myeloperoxidase (anti-MPO; red) and nuclear counterstain DAPI (blue) in the lung of experimental group of mice. Representative images are shown at 20X magnification, scale bar; 100 μm.

**Figure S5. Gating strategy for multi-color flow cytometry analysis of eosinophil and neutrophil population.**

**Supplementary Reference**

1. Nazaroff CD, LeSuer WE, Masuda MY, Pyon G, Lacy P, Jacobsen EA. Assessment of Lung Eosinophils In Situ Using Immunohistological Staining. *Methods Mol Biol.* 2021;2223:237-266.
