# Supplementary figures and images for: Mast cell MrgprB2 in neuroimmune interaction in IgE-mediated airway inflammation and its modulation by β-arrestin2
Source: Front Immunol. 2024 Oct 17;15:1470016. doi: 10.3389/fimmu.2024.1470016 (PMC11524863; doi:10.3389/fimmu.2024.1470016)

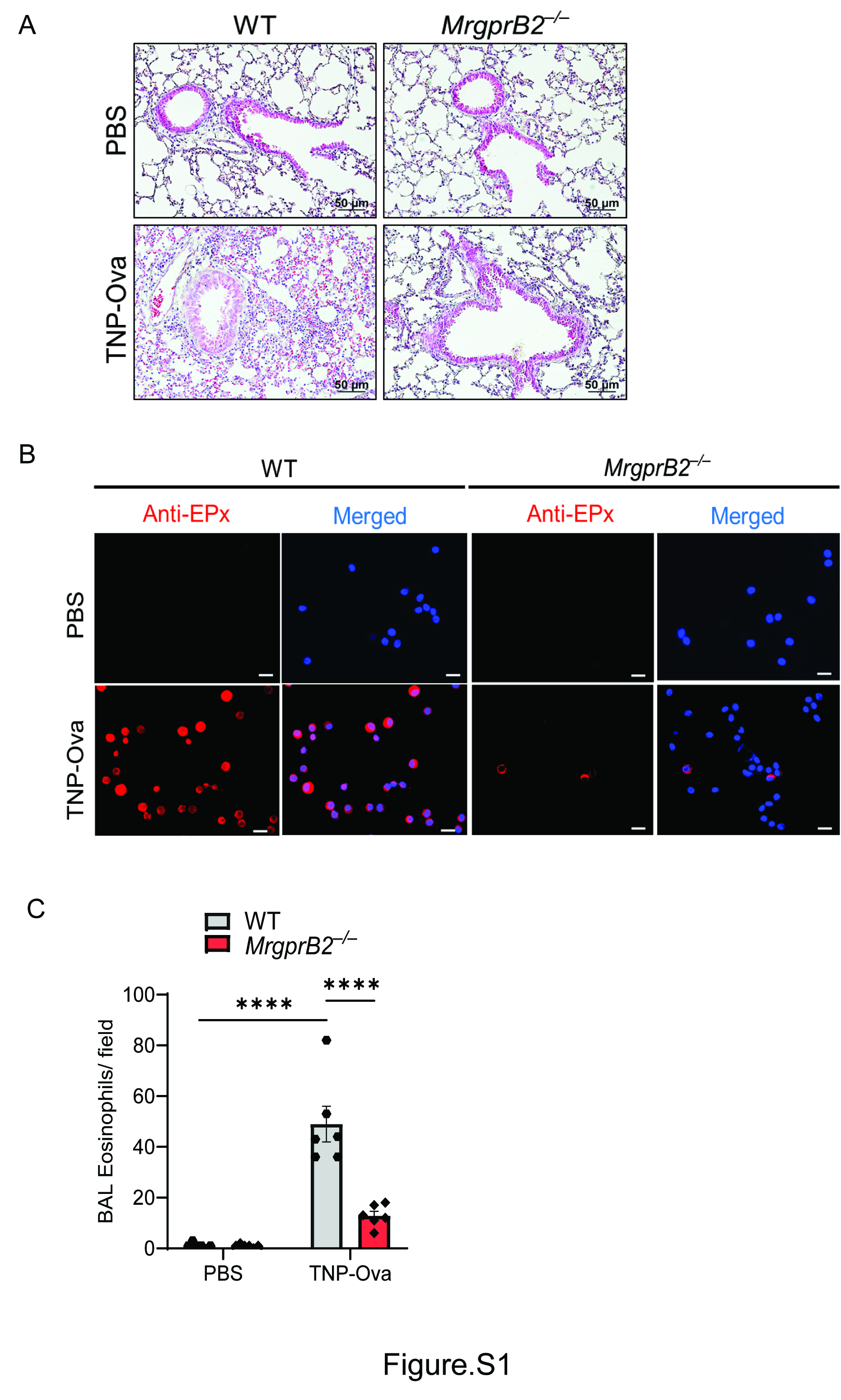

Supplement: Supplementary file 2 [file Image1.tif]

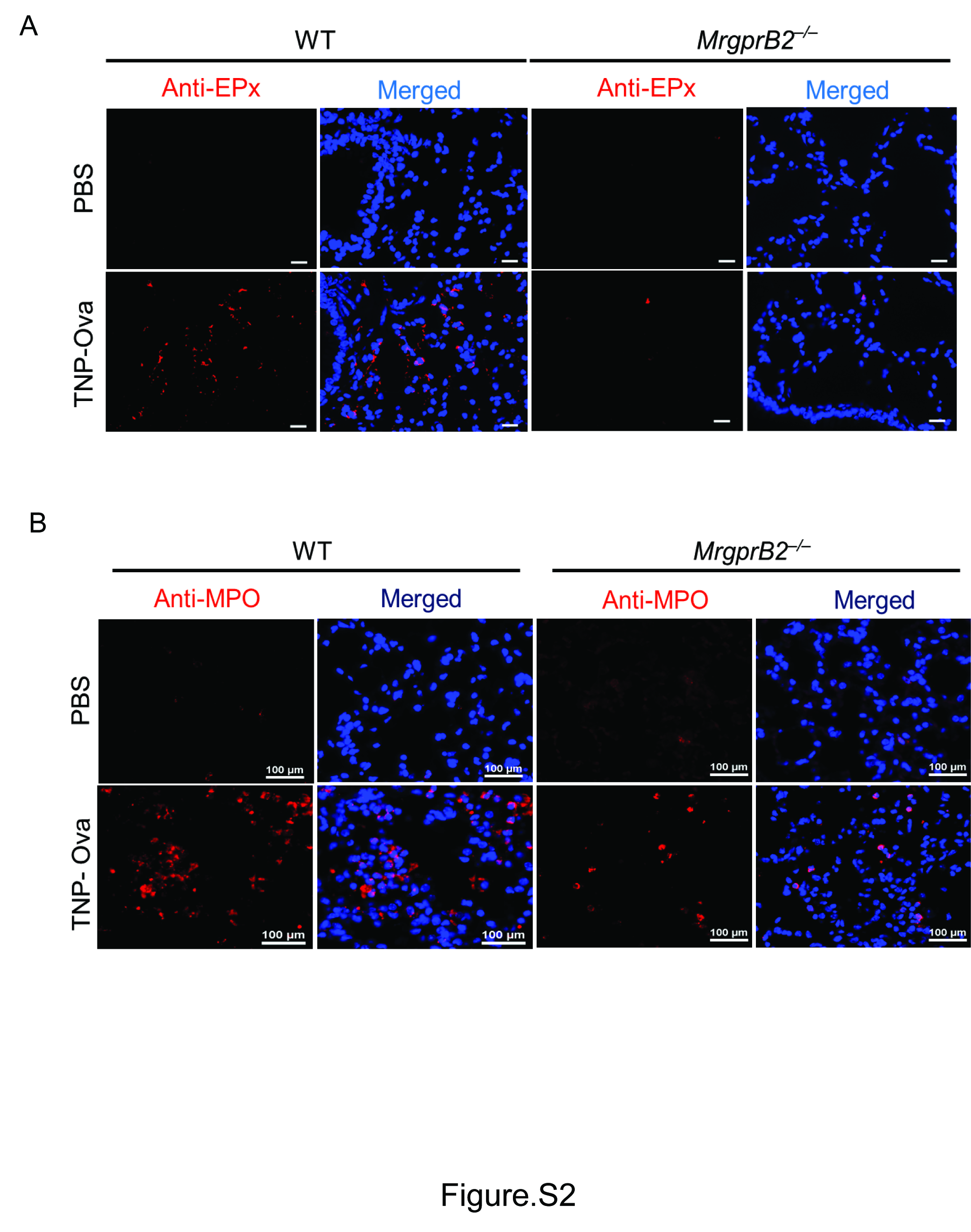

Supplement: Supplementary file 3 [file Image2.tif]

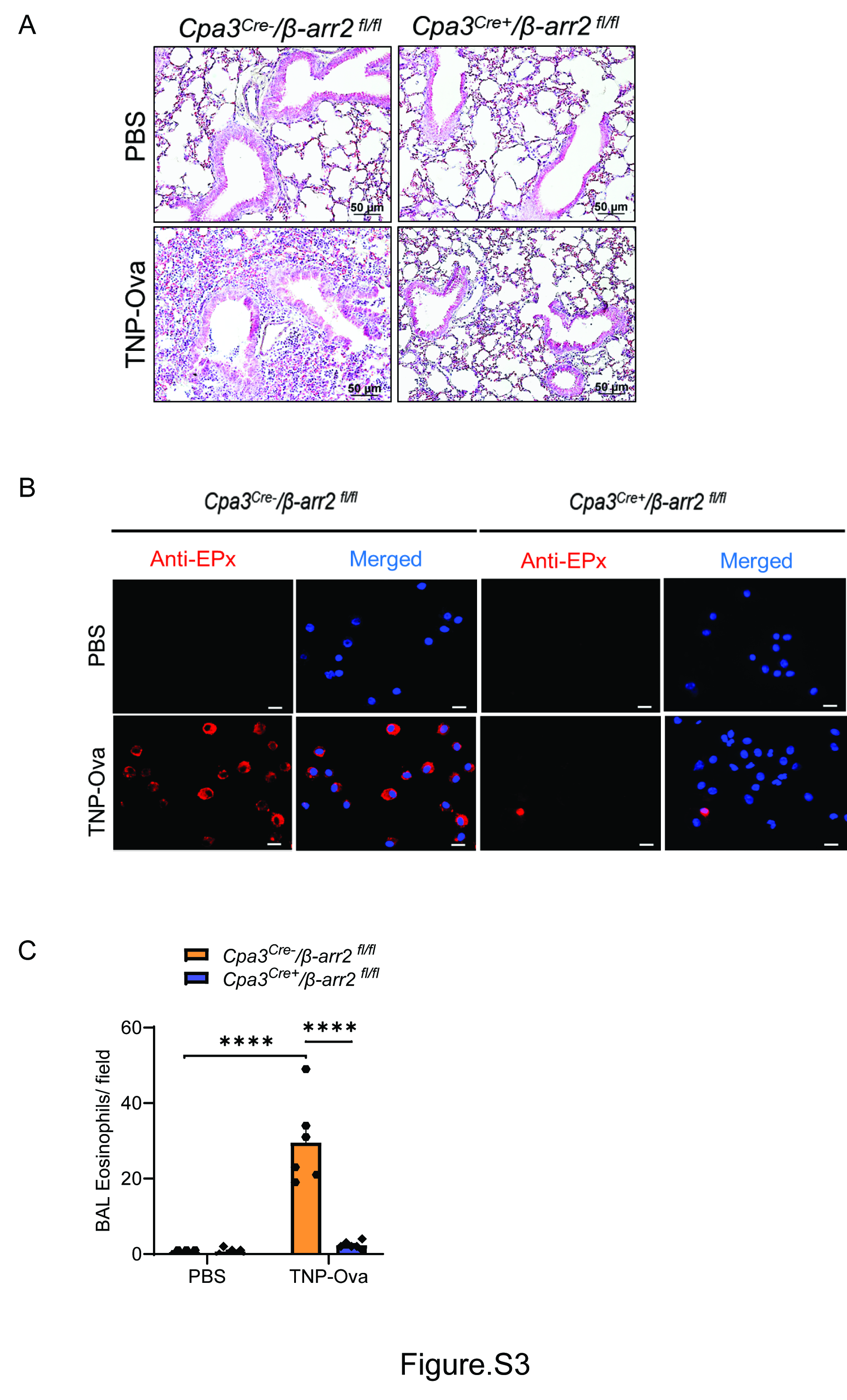

Supplement: Supplementary file 4 [file Image3.tif]

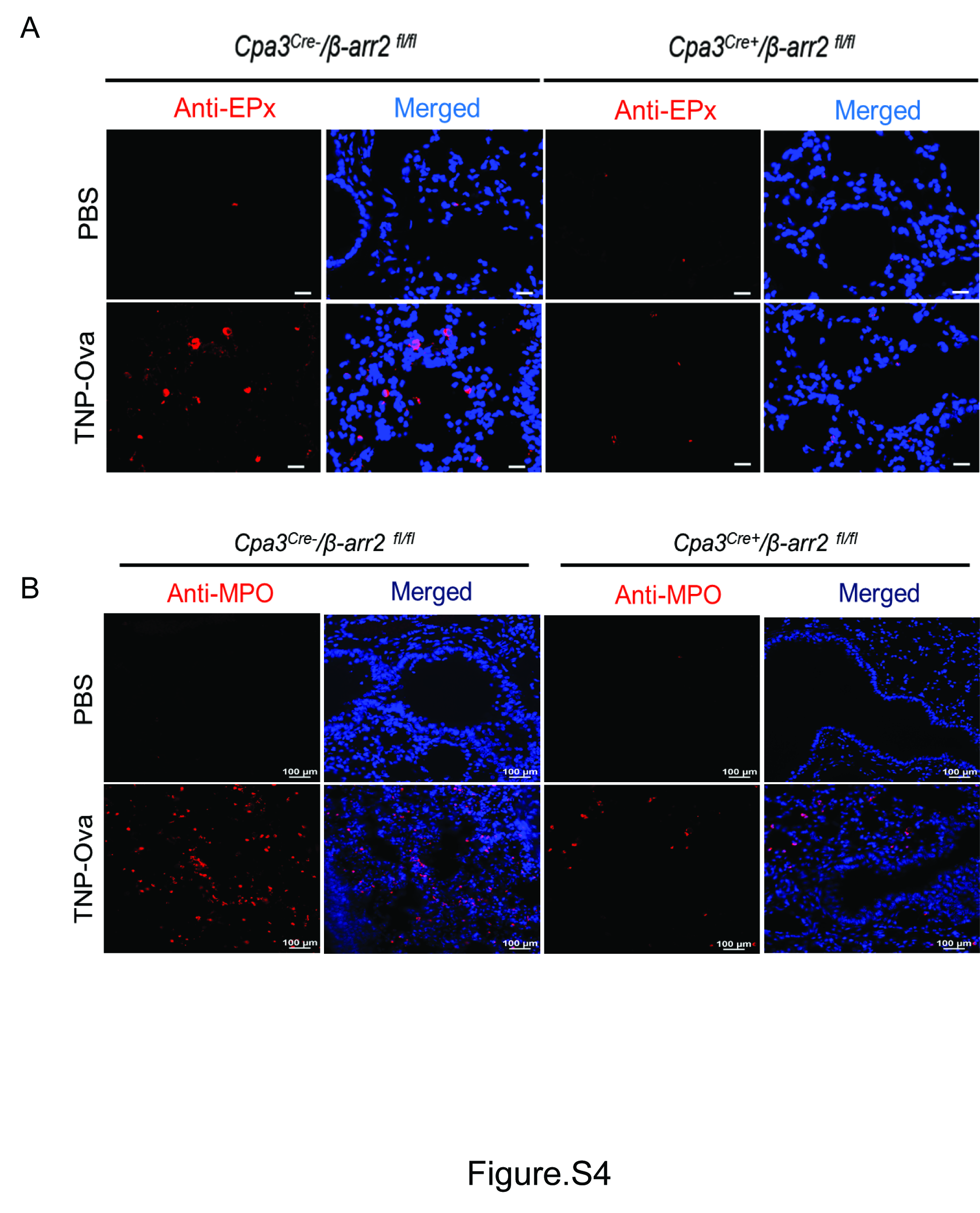

Supplement: Supplementary file 5 [file Image4.tif]

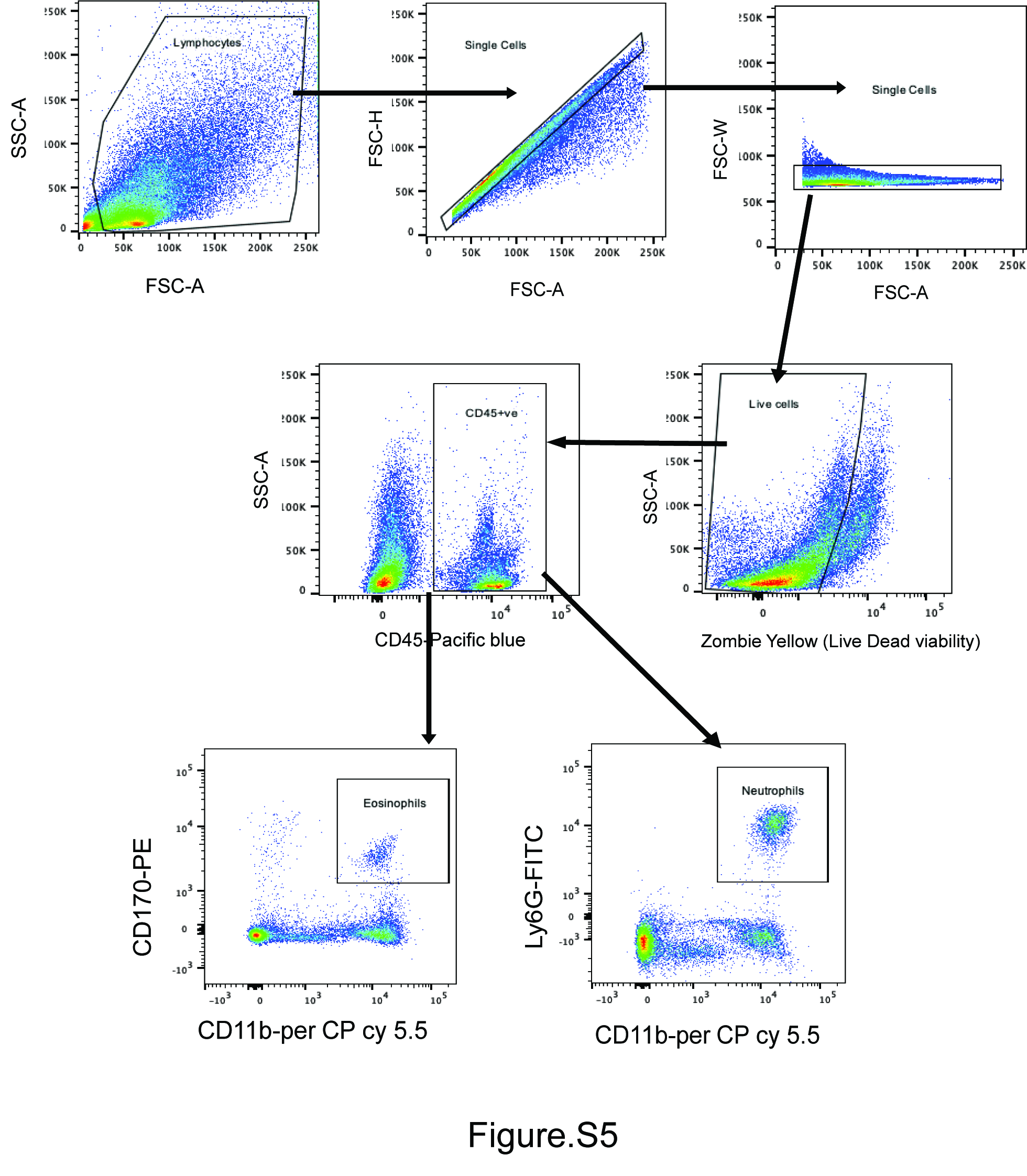

Supplement: Supplementary file 6 [file Image5.tif]
